# Supplementary material for: Perinatal care in Western Uganda: Prevalence and factors associated with appropriate care among women attending three district hospitals
Source: PLoS One. 2022 May 31;17(5):e0267015. doi: 10.1371/journal.pone.0267015 (PMC9154186; doi:10.1371/journal.pone.0267015)
Supplement: S1 Appendix — (DOC) [file pone.0267015.s001.doc]

**Appendix IV: Data Extraction Tool**

| **Date: ____________________**  **District : _________________________**  **Facility Name : _______________________________________**  **Interviewer Name: __________________________________________** |
| --- |
| **Questionnaire Code: / __ / __ / __ / __ / __ /** |
| **Patient Code : / __ / __ / __ / __ / __ /** |

**SECTION A: GENERAL INFORMATION**

The research team shall interview women for this information before their records are reviewed.

| Question | Response |
| --- | --- |
| 1. What is your age? | _____________ |
| 1. What is your parity (number of children including those who died and miscarriages)? | ______________ |
| 1. What is your highest level of education? | 1. No education 2. Primary level 3. Secondary level 4. Tertiary level |
| 1. What is your marital status? | 1. Married 2. Single 3. Separated 4. Widowed/widower 5. Others, specify ________ |
| 1. What kind of work do you do for a living? | 1. Formal employment 2. Farming 3. Casual Labourer 4. Business/Petty traders 5. Others, specify ___________ |
| 1. What is your monthly income? *(For farmers who get seasonal income, the income earned will be divided by the number of months)* | ________________________ |

**SECTION B: MANAGEMENT AND INFORMATION CONTINUITY**

For this section, the research team will review the records of perinatal mothers who were interviewed and fill in. Use a Tick (√) to mean “Yes” and Cross (X) to mean “No”

Questions 7 - 12 will refer to antenatal care records for the mother during the pregnancy period

| Question | Response |
| --- | --- |
| 1. Does the mother have an antenatal care record? | 1. Yes 2. No |
| 1. If yes, what kind of record? | 1. Mother child health passport 2. Antenatal card 3. Exercise book |
| 1. Are all areas in the mother’s record filled in? | 1. Yes 2. No |
| 1. According to the antenatal card, how many visits did the mother have? | ______________________ |
| 1. At how many weeks did the mother start antenatal care? | ________________________ |

1. According to the antenatal card/mother baby passport, what care did the mother receive and at how many weeks did she receive the care?

| Care aspect | Whether care was received or not | How often care was received (insert number of times) | At what weeks care was received |
| --- | --- | --- | --- |
| - 1. Received iron tablets for prevention of anaemia |  |  |  |
| - 1. Received Fansidar for prevention of malaria |  |  |  |
| - 1. Received TT for prevention of tetanus |  |  |  |
| - 1. Received health education |  |  |  |
| - 1. Had a hemoglobin (Hb) test done |  |  |  |
| - 1. Had her blood tested for syphilis |  |  |  |
| - 1. Had her blood tested for HIV |  |  |  |
| - 1. Had her urine tested for glucose and proteins |  |  |  |
| - 1. Had an ultrasound scan done |  |  |  |
| - 1. Fetal status assessed (fetal heart rate, fetal lie, fetal position) |  |  |  |
| - 1. Maternal health status assessed (BP, weight, pallor) |  |  |  |
| - 1. Pregnancy progress assessed (fundal height) |  |  |  |

Questions 13 - 17 will refer to the labor and delivery records for the mother

| Questions | Response |
| --- | --- |
| 1. Does the mother have a record indicating care given in labour? | 1. Yes 2. No |
| - 1. If yes, what kind of record? | 1. Patient file 2. Exercise book |
| 1. Does the mother have a partograph? | 1. Yes 2. No |
| - 1. Is the partograph completely filled? | 1. Yes 2. No |
| 1. What was the cervical dilatation on admission? | _________________ |
| 1. What was the cervical dilatation at the time partograph was initiated? | __________________ |

1. According to the partograph, what care did the mother receive and what was the frequency of the care?

| Care aspect | Whether care was received or not | How often care was received (insert frequency) |
| --- | --- | --- |
| - 1. Assessed fetal status (feta heart rate) |  |  |
| - 1. Assessed cervical dilation |  |  |
| - 1. Assessed descent of the presenting part |  |  |
| - 1. Assessed contractions during labour |  |  |
| - 1. Assessed vital signs (BP and PR) |  |  |

Questions 18 - 20 will refer to the postnatal records for the mother

| Question | Response |
| --- | --- |
| 1. Does the mother have any record of postnatal care received? |  |
| 1. How many times has the mother been examined from the time of delivery? |  |

1. According to the postnatal record, what care did the mother receive within the 24hours after delivery?

| Care aspect | Whether care was received or not |
| --- | --- |
| - 1. Assessed uterine contraction |  |
| - 1. Assessed vaginal bleeding |  |
| - 1. Assessed fundal height |  |
| - 1. Assessed vital signs (BP, PR, Temperature) |  |
| - 1. Assessed urine voiding |  |
